# Supplementary material for: Oral and plasma microbiome in the context of acute febrile illness
Source: medRxiv. 2026 Apr 20:2026.04.16.26351042. Preprint. [Version 1] doi: 10.64898/2026.04.16.26351042 (PMC13131847; doi:10.64898/2026.04.16.26351042)

**Oral and plasma microbiome in the context of acute febrile illness**

Mouhamad Sy^1,2^, Tolla Ndiaye^2,3^, Ritika Thakur^1^, Amy Gaye^2^, Zoe C. Levine^4,5^, Bassirou Ngom^2^, Karina L. Bellavia^1^, David Firer^1^, Mariama Toure^2^, Ibrahima M. Ndiaye^2^, Younouss Diedhiou^2^, Amadou M. Mbaye^2^, Jules F. Gomis^2^, Katherine C. DeRuff^5^, Awa B. Deme^2^, Mouhamadou Ndiaye^2,6^, Aida S. Badiane^2,6^, Marietou Faye Paye^5^, Pardis C. Sabeti^5,7,8,9^, Daouda Ndiaye^2,6#^, Katherine J. Siddle^1*#^.

1. Department of Molecular Microbiology and Immunology, Brown University, Providence, RI, USA.
2. International Research and Training Center for Applied Genomics and Health Surveillance (CIGASS) at UCAD, Dakar, Senegal.
3. Department of Microbiology and Immunology, Columbia University Irving Medical Center, NY, USA.
4. Harvard/MIT MD-PhD Program, Boston, MA, USA
5. Broad Institute of MIT and Harvard, Cambridge, MA, United States.
6. Department of Parasitology, Faculty of Medicine, University Cheikh Anta Diop, Dakar, Senegal.
7. Department of Organismic and Evolutionary Biology, Harvard University, Cambridge, MA 02138, USA.
8. Department of Immunology and Infectious Diseases, Harvard T.H. Chan School of Public Health, Harvard University, Boston, MA 02115, USA.
9. Howard Hughes Medical Institute, Chevy Chase, MD 20815, USA.

* address correspondence to [katherine_siddle@brown.edu](mailto:katherine_siddle@brown.edu)

# these authors jointly supervised this work

##

##

##

## **Supplementary materials**

# Contents

**Supplementary Figures**

**Supplementary Figure 1:** Sequencing depth and rarefaction curves

**Supplementary Figure 2:** Concordance across metagenomic classifiers

**Supplementary Figure 3:** Inter-individual variability for oral and plasma samples

**Supplementary Figure 4:** Top 50 species detected by Kraken2 and sourmash

**Supplementary Figure 5:** 16S rRNA and metatranscriptomic species abundance estimates

**Supplementary Figure 6:** Per-species abundance estimates between methods methods

**Supplementary Figure 7:** Correlation between 16S and metatranscriptomic species abundance

**Supplementary Figure 8:** Hybrid capture sequencing depth

**Supplementary Figure 9:** Phylogenetic investigation of redondoviruses

**Supplementary Figure 10:** Oral microbiome diversity by covariates and infection

**Supplementary Figure 11:** VF detection by sample and species

**Supplementary Tables**

**Supplementary Table 1:** Contaminant list for filtering

**Supplementary Table 2:** Known pathogens participant demographic characteristics


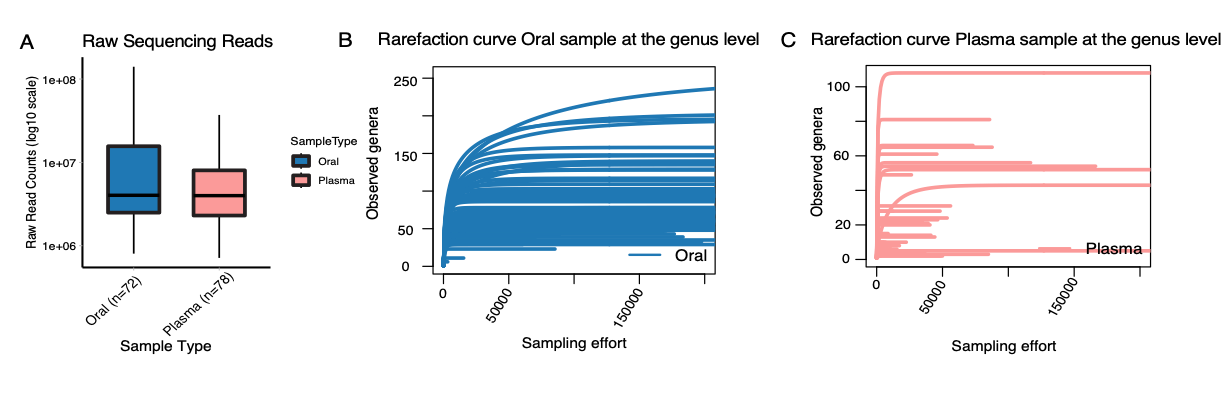


**Supplementary Figure 1 :** A) Sequencing depth, B) Rarefaction curve oral sample at the genus level, C) Rarefaction curve plasma sample at the genus level.


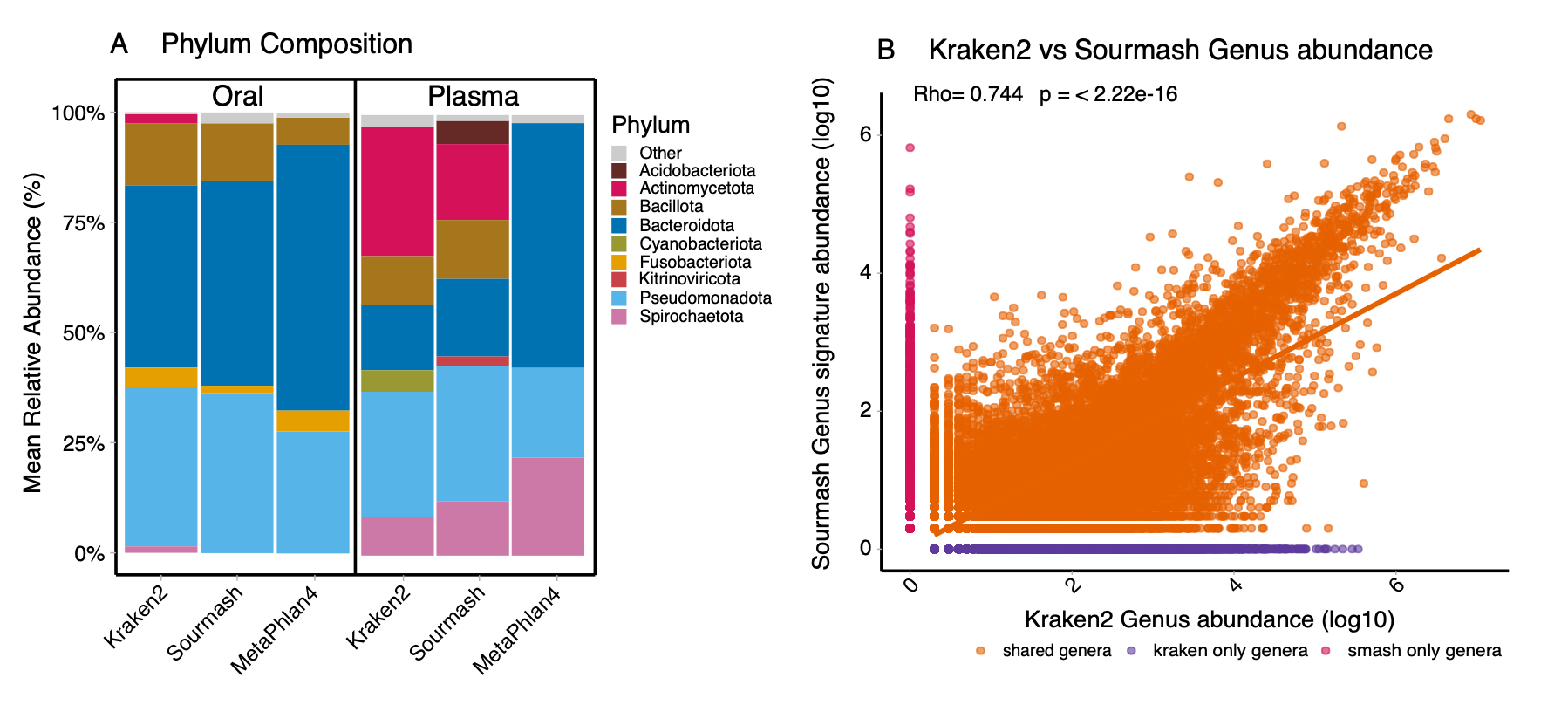


**Supplementary Figure 2:** Phylum mean relative abundance and correlation plots between Kraken2 and Sourmash.

**
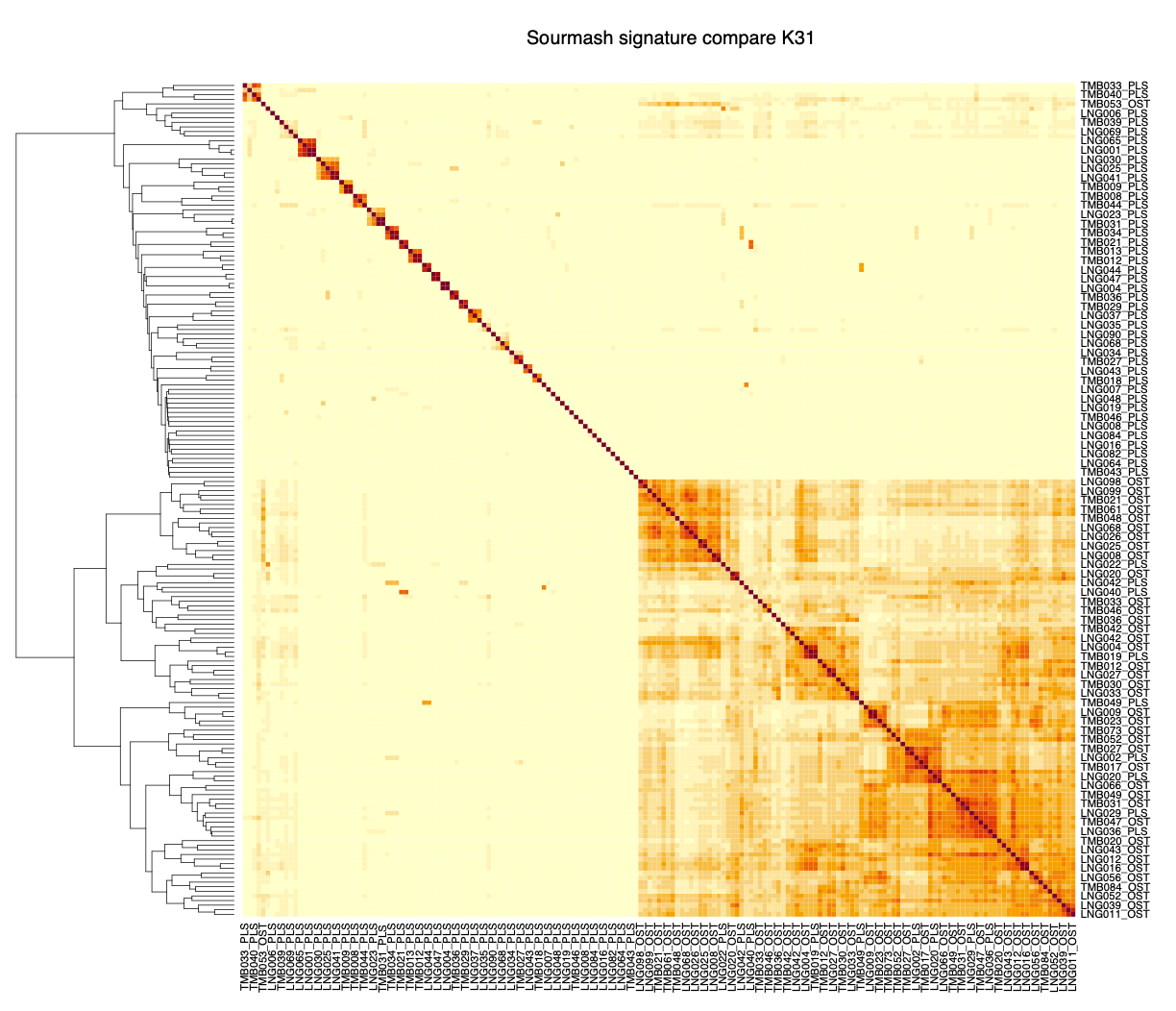
**

**Supplementary Figure 3:** Sourmash signature comparison (k-mer = 31). The dark red indicates high-similarity between samples.


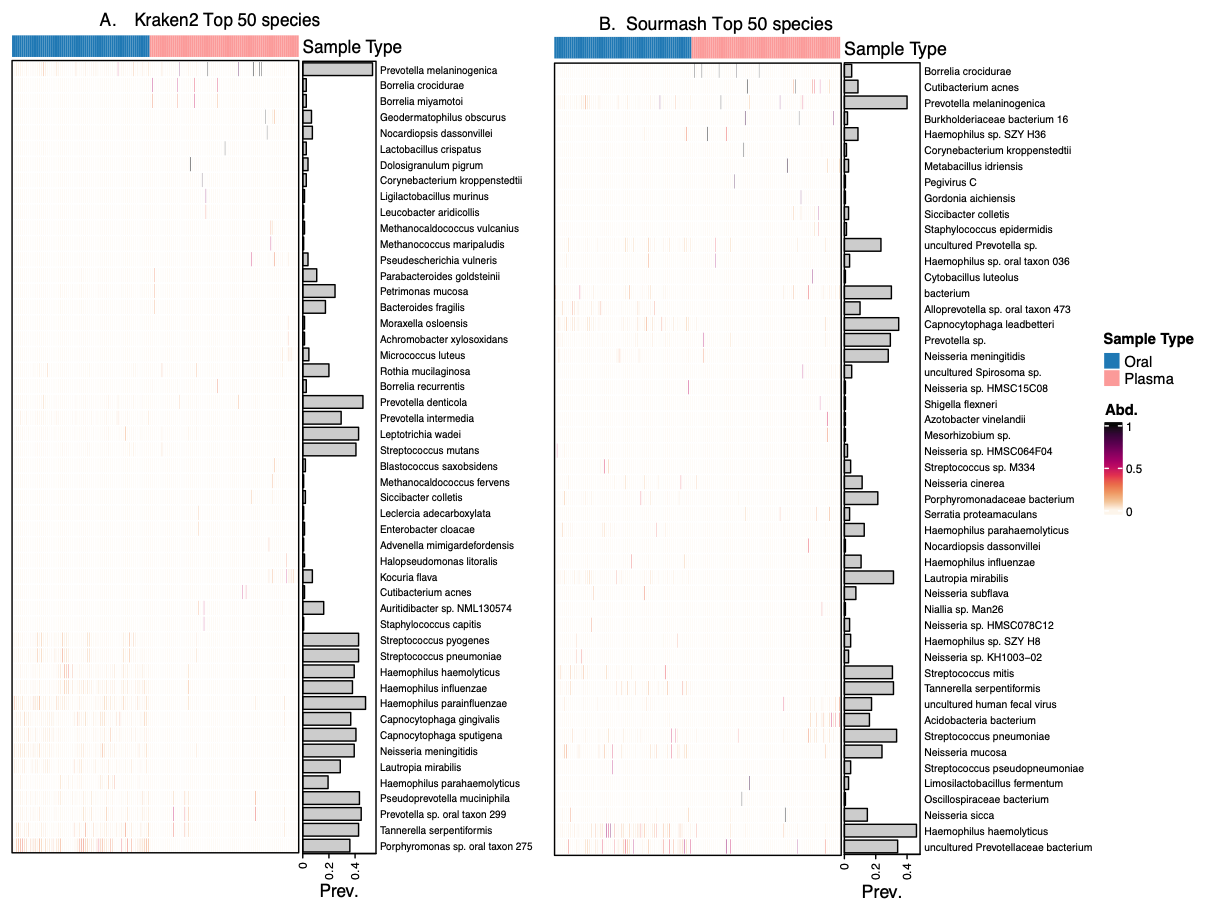


**Supplementary Figure 4** : Top 50 species detected by kraken2 (A) and Sourmash (B)


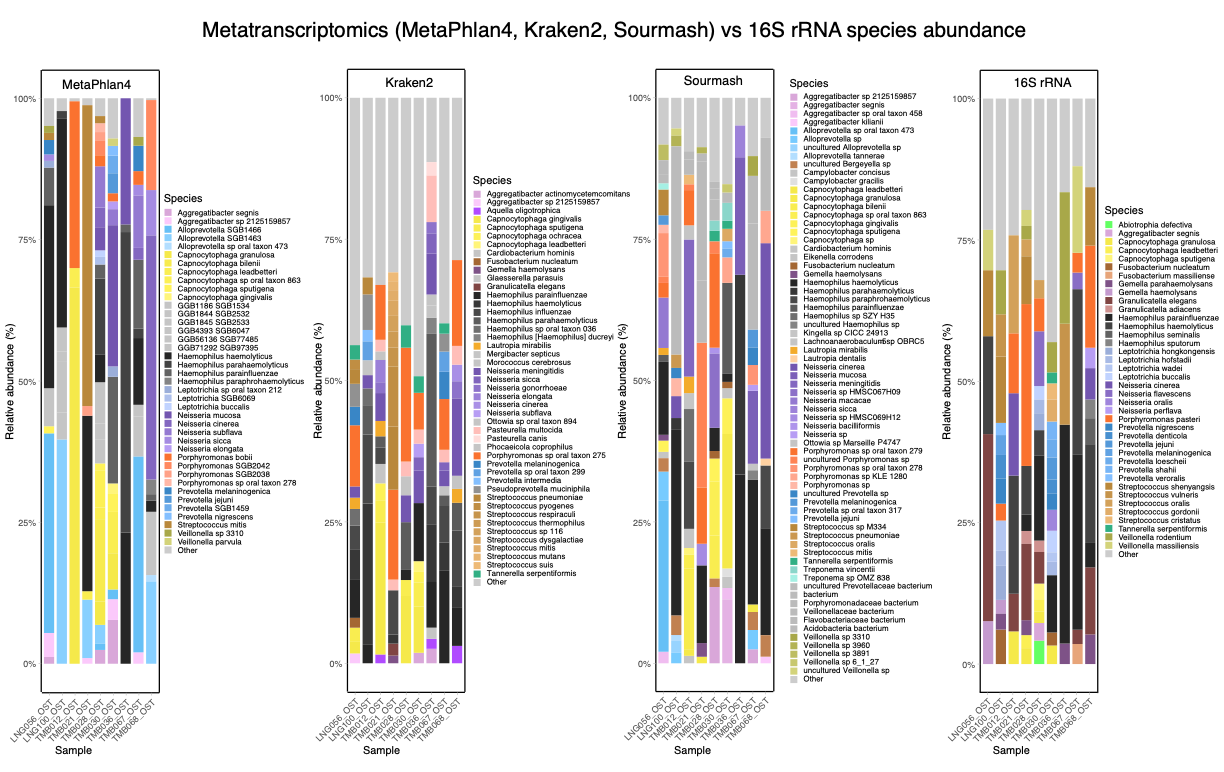
**Supplementary Figure 5** : 16S rRNA and metatranscriptomic species abundances
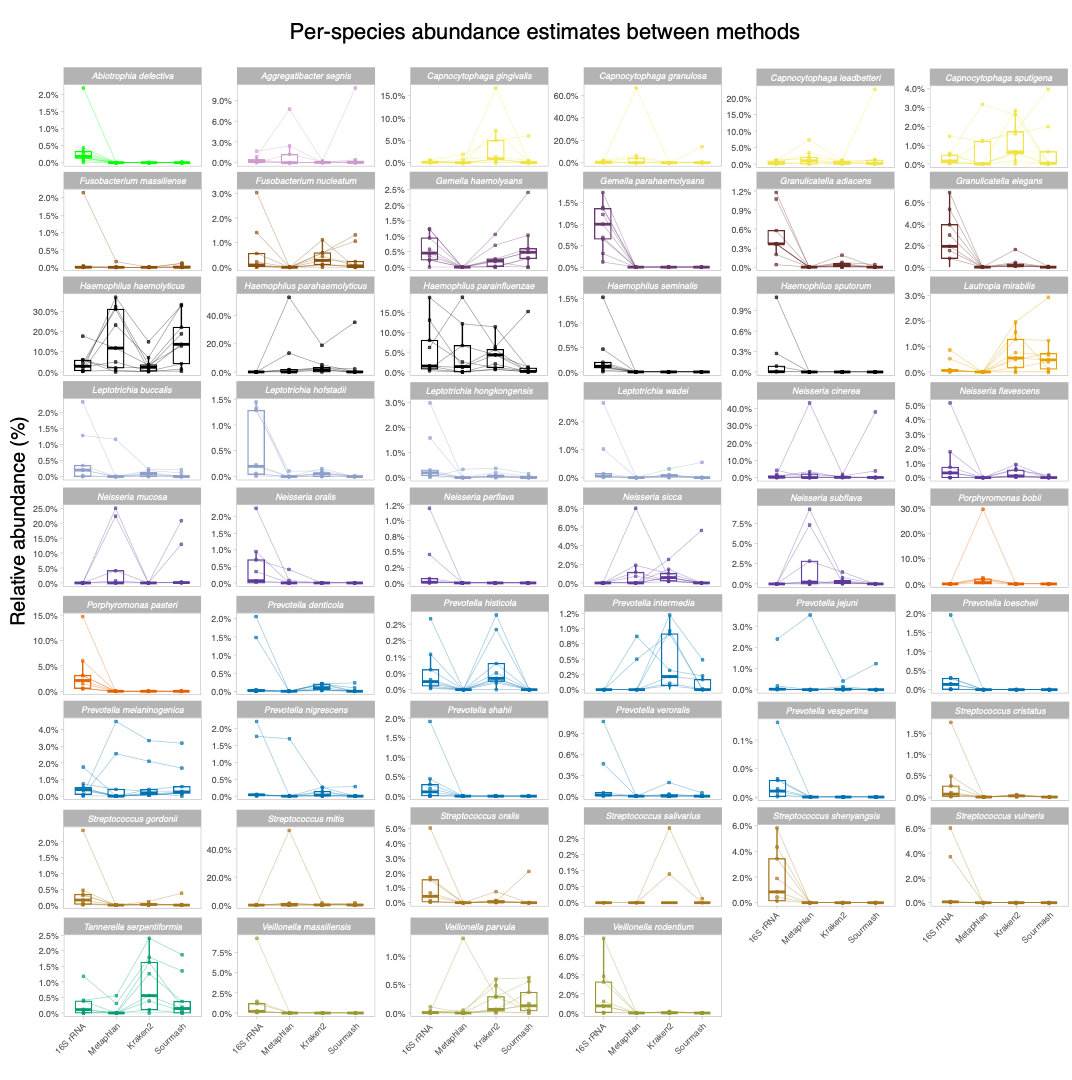


**Supplementary Figure 6** : 16S top species abundance compared to abundance found with metatranscriptomic analysis with kraken2, sourmash and Metaphlan4.


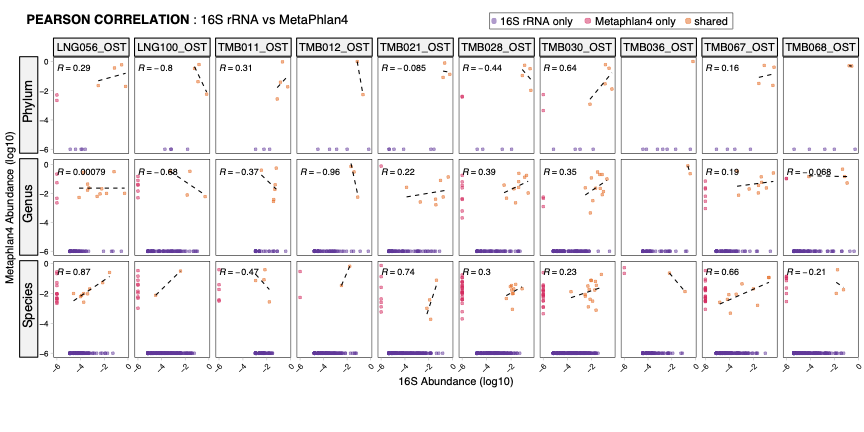


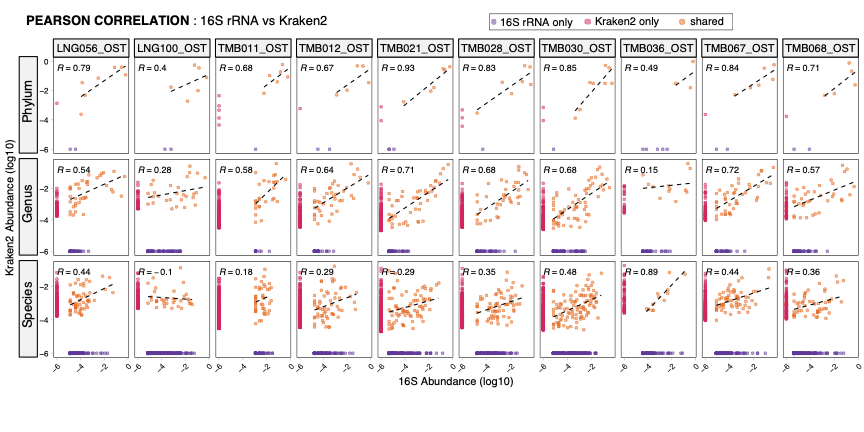


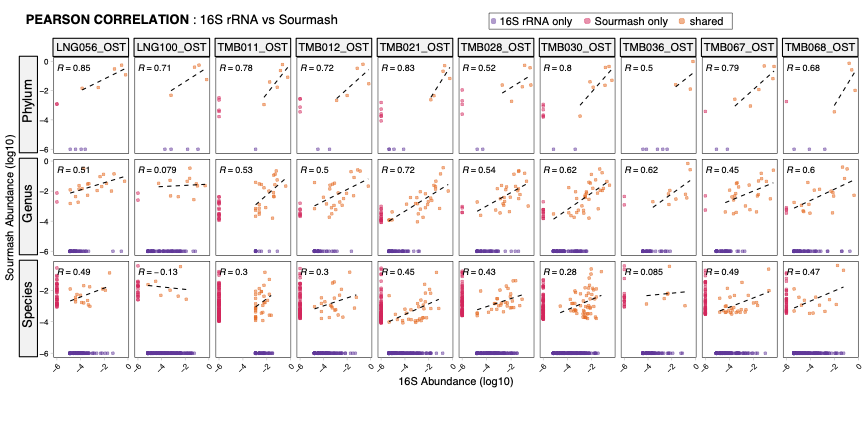


**Supplementary Figure 7** : Pearson correlation between 16S rRNA and Metatranscriptomic tools at the individual sample at the Phylum, Genus and Species level. Correlation analysis was performed only at the share taxon.


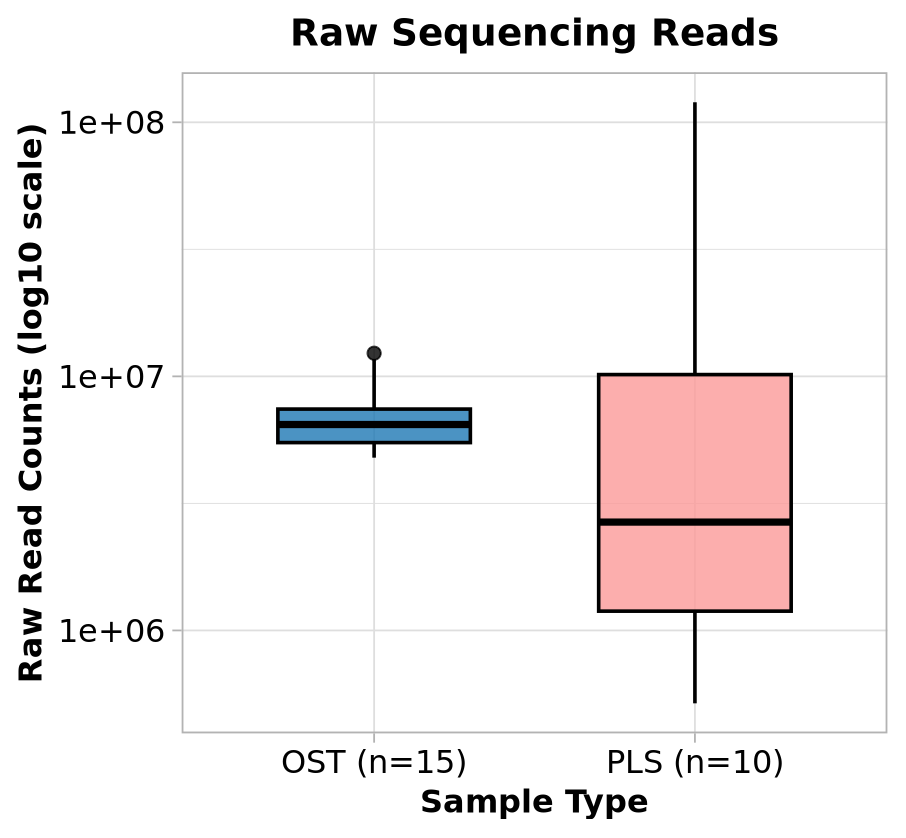


**Supplementary Figure 8**: Hybrid capture sequencing depth. Boxplot showing raw read depth for oral (OST, N=15) and Plasma (PLS, N=10) samples after hybrid capture enrichment with pan-viral enrichment probes.


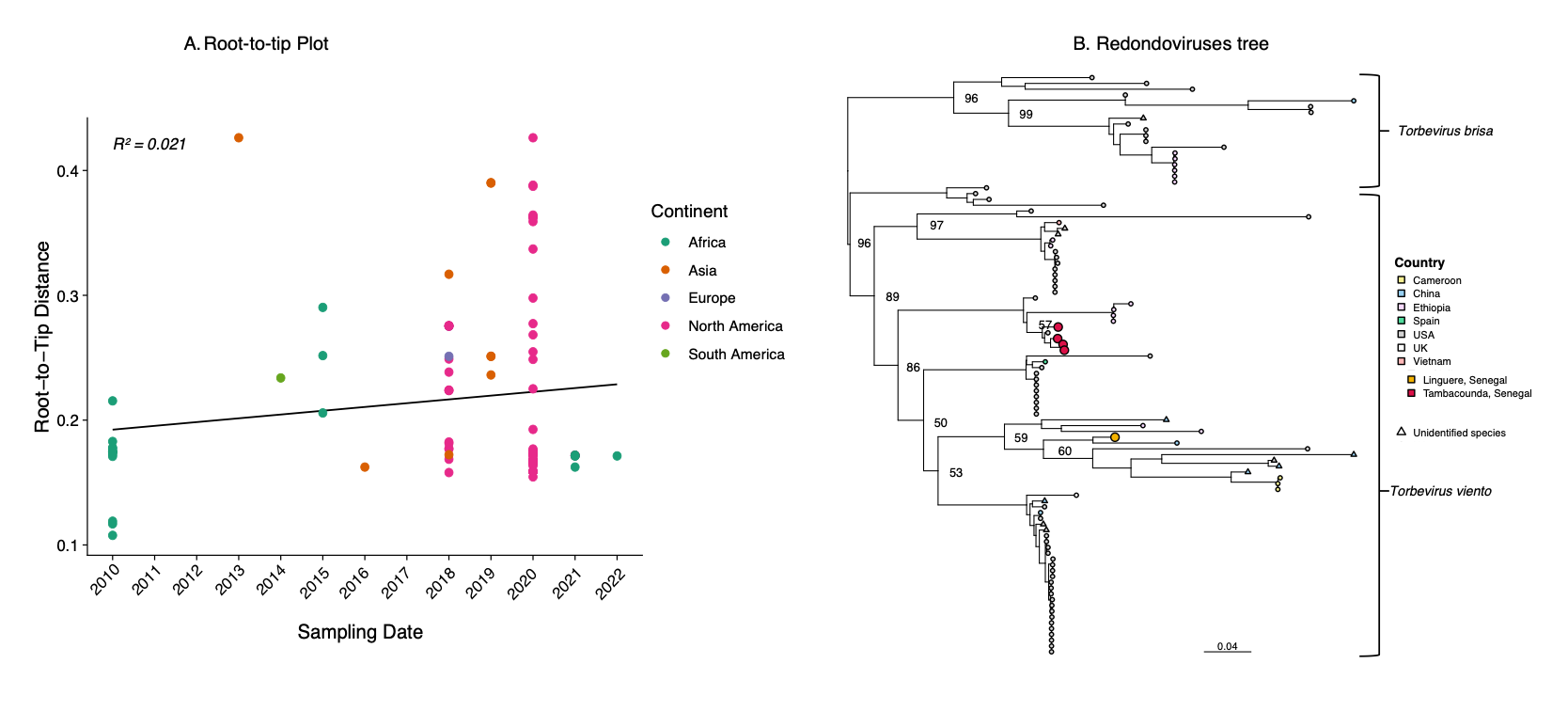


**Supplementary Figure 9:** Phylogenetic investigation of redondoviruses. A. Root-to-tip plot of all vientovirus samples. B. maximum likelihood tree of the viral Cap gene.

**
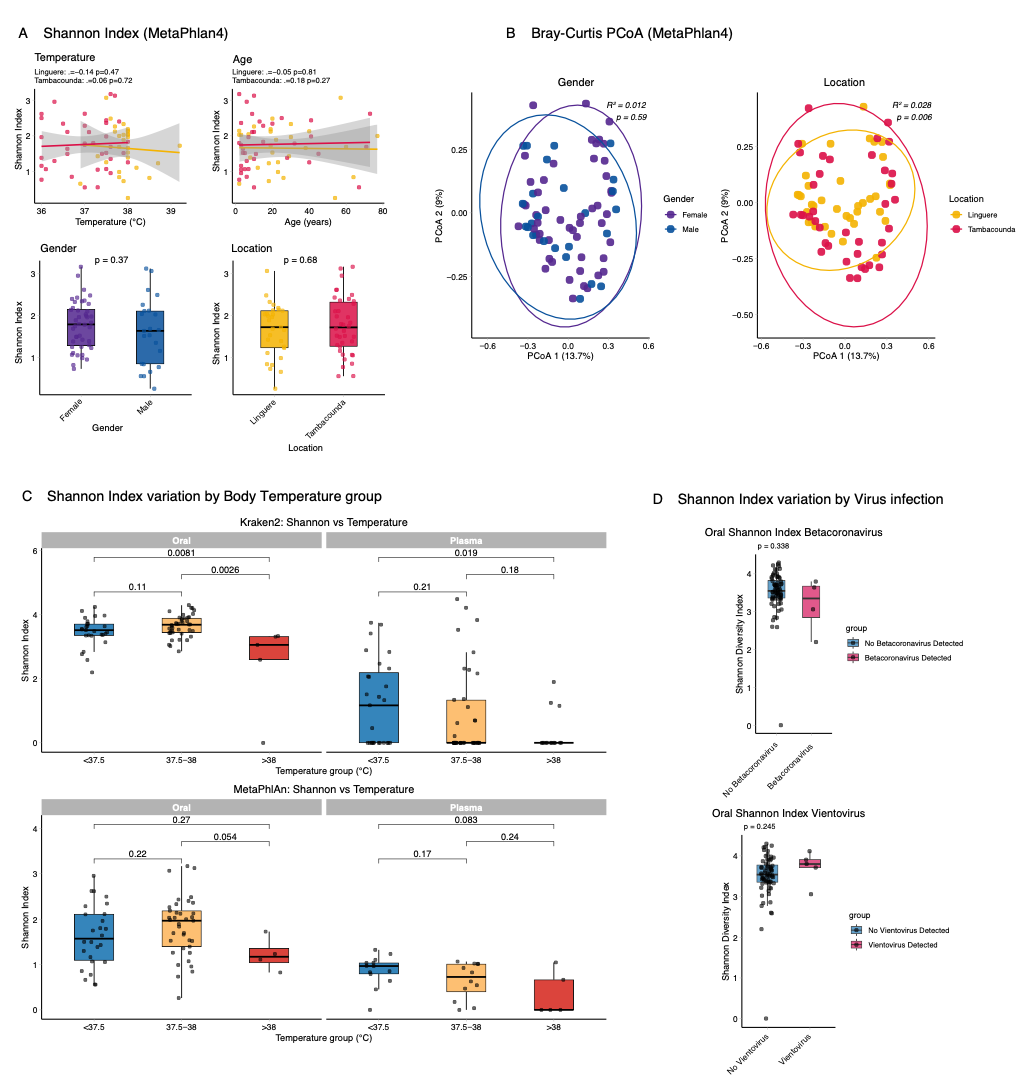
**

**Supplementary Figure 10:** A) Oral sample Shannon Index with MetaPhlan for Temperature, Age, Gender and Location, B) Oral sample Bray-Curtis principal coordinate analysis (PCoA) plot depicting differences in microbial composition for Gender and Location with MetaPhlan, C) boxplots of Shannon Index for different body temperature groupings using Kraken2 and MetaPhlan abundance estimates for plasma and oral sample, D) Shannon index comparison between group infected with SARS-CoV-2 and Vientovirus and group with no viral infection.


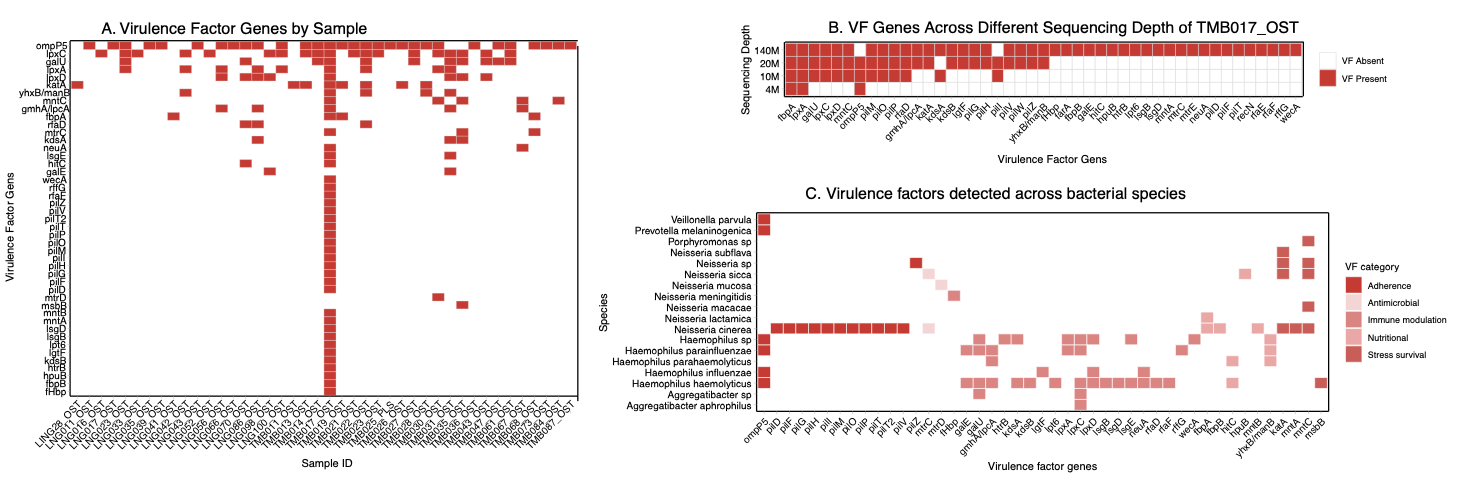


**Supplementary Figure 11:** A) Virulence factor (VF), B) VF detected in sample TMB017_OST across different sequencing depth C) Bacterial Species with VF Genes.

**Supplementary Table 1:** Contaminant list for filtering


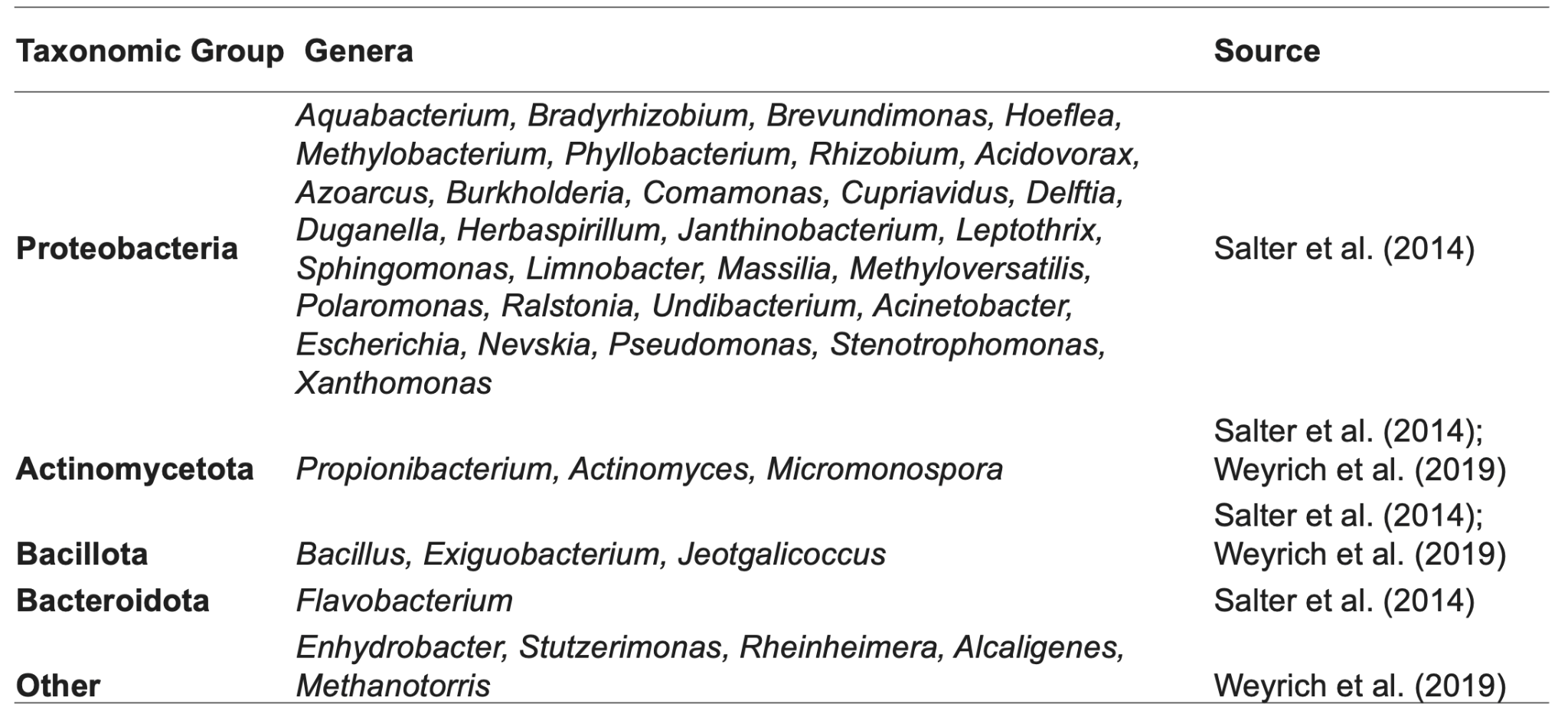


**Supplementary Table 2:** Known pathogens participant demographic characteristics


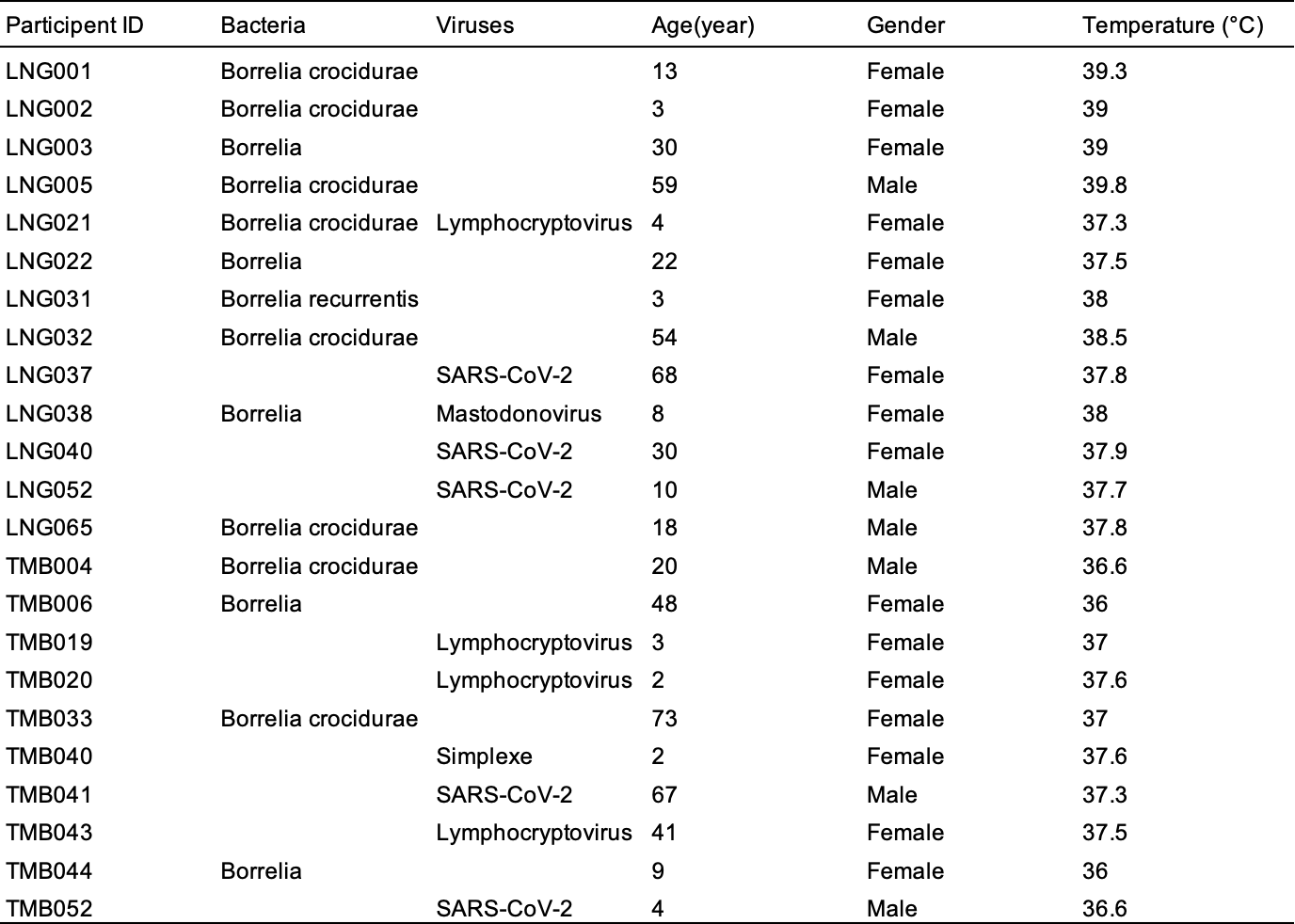

Supplement: Supplement 1 [file media-1.docx]
